# Biocontrol Agents and Natural Feed Supplements as a Safe and Cost-Effective Way for Preventing Health Ailments Provoked by Mycotoxins

**DOI:** 10.3390/foods14111960

**Published:** 2025-05-31

**Authors:** Stoycho D. Stoev

**Affiliations:** Department of General and Clinical Pathology, Faculty of Veterinary Medicine, Trakia University, 6000 Stara Zagora, Bulgaria; stoycho.stoev@trakia-uni.bg

**Keywords:** food security, mycotoxins, foodborne mycotoxicoses, prevention, management of the risk, natural feed supplements

## Abstract

The relationships between mycotoxins content in food commodities or feedstuffs and the foodborne diseases is well known. So far, the available data mainly include chemical methods of mycotoxins decontamination for agricultural commodities or raw materials, including mycotoxin binders. Therefore, the possible use of some natural and cost-effective supplements such as herbs, fungi, microorganisms, or plants with powerful and safe protection against mycotoxin-induced health ailments is the main subject of this review paper. Various antagonistic microorganisms or yeast with fungicidal properties, as well as some herbs or plants that suppress fungal development and the subsequent production of target mycotoxins and/or have protective effect against mycotoxins, are deeply studied in the literature, and practical suggestions are given in this regard. The protection by degradation, biotransformation, or binding of mycotoxins by using natural additives such as herbs or plants to feedstuffs or foods has also been thoroughly investigated and analyzed as a possible approach for ameliorating the target adverse effects of mycotoxins. Possible beneficial dietary changes have also been studied to potentially alleviate mycotoxin toxicity. Practical advice are provided for possible application of the same natural supplements in real-life practice for combating mycotoxin-induced health ailments. Natural feed supplements and bioactive compounds appeared to be safe emerging approaches to preventing health ailments caused by mycotoxins. However, the available data mainly address some in vitro studies, and more in vivo experiments are necessary for introducing such approaches in the real-life practice or industry. Generally, target herbal supplements, antioxidants, or polyenzyme complements could be used as powerful protectors in addition to natural mycotoxin binders. Bioactive agents and enzymatic degradation are reported to be very successful in regard to PAT and OTA, whereas antagonistic microorganisms/fungi/yeasts have a successful application against AFs and PAT-producing fungi.

## 1. Introduction

Mycotoxins are metabolites produced by fungi and are often contaminants of feedstuffs and food products, thus presenting a serious danger to animal and human health. Mycotoxin-contaminated food and feed are responsible for many foodborne diseases and health problems in both animals and humans. Such foodborne diseases are often observed in developing countries, where food/feed control is not very strict due to lower standards of food quality or the absence of adequate regulations [1]. The fungi invade cereals in the field or after the harvest, and such an invasion is often unavoidable depending on target environmental conditions, such as rain at the time of harvest or undesirable conditions during the storage of grain or feedstuffs. Therefore, the contamination of food/feed with mycotoxins is often reported in various countries, and such contamination usually involves multiple mycotoxins because a single fungus or several fungi usually produce many different mycotoxins in a single food commodity or grain. Such large-scale mycotoxins contamination of feeds/foods usually leads to serious hazards to animal/human health and a significant economic burden [2,3]. Many mycotoxins are currently reported as natural contaminants, but only approximately 12 are known to pose serious health hazards to animals and humans, including aflatoxins (AFs), with aflatoxin B1 (AFB1) and aflatoxin M1 (AFM1) being the most toxic, ochratoxin A (OTA), zearalenone (ZEA), T-2 and HT-2 toxins, patulin (PAT), deoxynivalenol (DON), fumonisins (FUMs), with fumonisin B1 (FB1) being the most toxic, nivalenol (NIV), diacetoscirpenol (DAS), and ergot alkaloids. The same mycotoxins are often contaminants of human food or animal feedstuffs at dangerous concentrations, being responsible for foodborne diseases or health ailments [1,2,3]. Some mycotoxins are also found to be frequent contaminants in animal products such as milk, meat, and eggs when ingested via feedstuffs [4].

**Figure 1:**
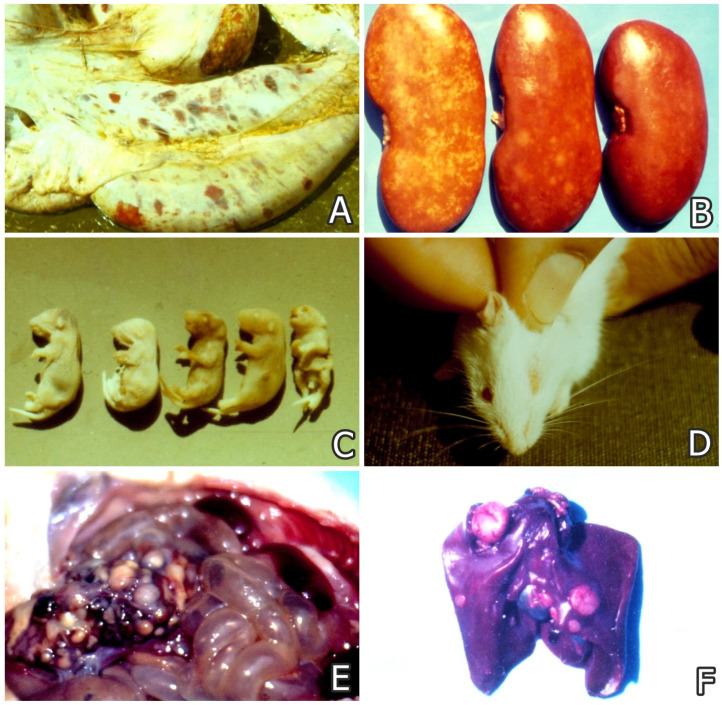
(**A**) Spontaneous case of stachybotryotoxicosis in cattle showing hemorrhages and necroses visible on the serosa and under the serosa of the abomasus [2]. (**B**) Spontaneous case of mycotoxic porcine nephropathy identified at the slaughterhouse in Bulgaria showing mottled and enlarged kidneys from pigs at 6–8 months of age (left) and normal appearance of kidneys of pig of the same age (right) [2]. (**C**) Malformations in newborn mice whose mothers were treated with 20 ppm OTA via the feed between day 7 and day 12 of pregnancy: astomia and anophthalmia (2 offspring on left), normal fetus (2 offspring in center), and spina bifida, incl. maxillary hypoplasia and facial cleft (1 offspring on right). (**D**) Monophthalmia in a mouse, whose mother was treated with 10 ppm OTA via the feed up to day 8 of pregnancy [11]. (**E**) Adenocarcinoma in the intestine of a rat exposed to 10 ppm OTA in its diet for 19 months, showing large gray-white neoplastic foci on the intestinal serosa protruding significantly above its surface. (**F**) Adenocarcinoma in the liver of a male chick exposed to 5 ppm OTA in its diet for 10 months, showing gray-white neoplasia on the liver protruding above its surface [10,14].

Mycotoxin exposure of animals or poultry through diet causes health problems such as decreased weight gain, poor feed conversion, refusal of feed, foodborne diseases or ailments (Figure 1) [2,5], and some target disturbances in reproductive possibilities [6,7]. In recent years, biocontrol agents [8], in addition to biopesticides derived from microbial sources (viruses, bacteria and fungi) [9], have gained great popularity due to their persistence, non-toxic nature, and specific action against target fungal species producing mycotoxins.

The known harmful effects of mycotoxins are nephrotoxic (e.g., OTA and FB1) (Figure 1C), hepatotoxic (e.g., AFB1 and OTA), neurotoxic (e.g., FB1 and DON), estrogenic (e.g., ZEA and less DON) [5], immunosuppressive (e.g., OTA, AFB1, and T-2 toxin), teratogenic and genotoxic (e.g., OTA, AFB1, and T-2) (Figure 1C,D), or carcinogenic (e.g., AFB1, OTA, and FB1) (Figure 1E,F) [10,11,12,13,14,15,16]. In addition, some secondary microbial infections [17,18], more severe progression of some microbial diseases, or even parasitic diseases, due to the suppression of humoral or cell-mediated immune responses, are often reported in mycotoxin-compromised animals [2,5].

Therefore, it is necessary to employ new ways for safe mycotoxin decontamination by using natural feed additives [19,20] or organic binders and bioactive supplements in order to safely utilize mycotoxin-contaminated feeds and foods [21,22,23,24].

Foods or feedstuffs are often reported to be contaminated with multiple mycotoxins at low concentrations, which are within the accepted European requirements that are below the maximum permitted levels; however, such multi-mycotoxin contamination, even at low concentrations, could be dangerous for animals or humans, as a consequence of synergistic or additive interactions between target mycotoxins. Therefore, the toxicity of such low levels of target mycotoxin combinations must be carefully investigated [1]. The necessary hygiene control, risk assessment, and possible hazards to the health of animals and humans should be carefully investigated to define adequate preventive or protective measures under such circumstances.

The FAO has reported that nearly 25% of crops in the world are affected by mycotoxins each year, and losses of approximately 1 billion tons of food products are reported each year as a consequence of mycotoxin contamination [25]. Many people worldwide are constantly exposed to mycotoxins in food products such as milk, dairy products, meat, spices, coffee, beer, wine, and various cereal products [1,3,7]. Therefore, various types of economic or social losses have been reported, which are a consequence of disease and death among animals, health ailments in humans, higher costs of veterinary treatment and health services or medical care, reduction in animal growth and productivity, increased costs of mycotoxin control and preventive measures, increased costs of research and detoxification or elimination of mycotoxins, and losses due to scrapping feedstuffs or foods [26].

Considering that chemical methods for mycotoxin decontamination can deteriorate the nutrient value of feedstuffs or food commodities, their large-scale industrial use, except for ammoniation, is perceived as impractical and potentially harmful [27]. Unfortunately, ammoniation was also found to induce undesirable changes in the nutritional quality of feedstuffs, such as a decrease in sulfur- and lysine-containing amino acids [28]. In addition, aeration of feed after ammoniation is necessary for animals to accept it. Moreover, in some countries, the use of chemicals to decrease mycotoxin levels in juices is often not tolerated by food laws. In addition, the remaining residues of such chemical compounds may provoke undesirable side effects when ingested by animals and humans via feedstuffs or food [29,30].

The use of clays as nycotoxin binders at a large-scale industrial level is also controversial because of the deteriorating nutrient value of food commodities owing to the binding of some nutrients in addition to mycotoxins. On the other hand, most clays, such as kaolin, sepiolite, and bentonite, are rarely effective against most mycotoxins, such as OTA, T-2 toxins, FUMs, and DON [5,31,32,33], with the exclusion of AFs and PAT. Therefore, natural organic binders have also been suggested for the same purpose due to their efficacy against multiple mycotoxin contaminants, as reported in most cases. Natural organic binders are also highly biodegradable, preventing possible environmental contamination [34].

Obviously, natural feed additives, natural organic binders, and bioactive supplements can be used to safely utilize mycotoxin-containing grains, feedstuffs, or foods without any subsequent health problems for animals or humans [5,35,36].

This review aims to elucidate some safe and effective ways to eliminate the most dangerous mycotoxins to human and animal health that often contaminate agricultural commodities. A risk assessment and some safe ways of risk management of such contamination using natural feed supplements, bioactive substances, or biocontrol agents will be explored in the literature to define the efficacy of such measures in the safe utilization of feed/food. The possible use of such natural feed/nutritional supplements or probiotics in practice to reduce or eliminate the toxic effects of target mycotoxins will be investigated in depth.

## 2. Biological Methods of Protection Against Mycotoxin Contamination

Natural feed supplements can destroy the toxicity of mycotoxins through the action of target enzymes or live microorganisms, which participate in the detoxification or biotransformation of mycotoxins. Such supplements usually attract the attention of industrial feed producers because they propose a safe and promising strategy for reducing mycotoxin exposure, which is often achieved by decreasing the bioavailability of mycotoxins [5,35,36,37]. There are various kinds of biological additives that can be used to reduce feed contamination with mycotoxins and/or for mycotoxin degradation, including microbial/fungal antagonists and mycotoxin degradation by live or dead microorganisms. A decrease in mycotoxin contamination can also be achieved by cultured extracts of yeasts and some mycotoxins that destroy enzymes or proteins, all of which are non-toxic to animals and poultry and can be easily excreted from the body [38]. Biological methods for mycotoxin detoxification can usually preserve the flavor, organoleptic properties, and nutritional quality of treated food or forages and are safer and more cost-effective than other methods for mycotoxin decontamination [39].

### 2.1. Protection by Biodegradation, Biotransformation, or Binding of Mycotoxins

Detoxification of mycotoxins by degradation or biotransformation is a valuable modern strategy based on enzymatic or microbial methods of mycotoxin degradation. Some microbial enzymes or enzymatic preparations may be useful for such degradations. The protective properties of the gut or ruminal microbiota in some animals can be explained by the degradation of some mycotoxins into less toxic compounds or by a disturbance in the process of mycotoxin absorption by the same microbiota [40,41,42]. In this regard, some probiotics have been developed for mycotoxin detoxification and degradation using the same microbiota or digestive microflora of target animals [43].

The enzymatic degradation of mycotoxins has not been reported to be very useful in terms of AFs, FUMs, DON, and ZEA, and these mycotoxins are often present in beer produced from maize or wheat [44]. However, partial enzymatic degradation of OTA was observed, independent of the fact that OTA is a relatively stable mycotoxin in acidic or alkaline environments. A powder of the oyster mushroom *Pleurotus ostreatus* was recently found to have great potential to destroy OTA via simulation of in vitro gastrointestinal digestion in the presence or absence of ground feed or cornmeal. However, this powder was reported to be ineffective against ZEA [45] (Table 1) [39,45,46,47,48,49,50,51,52,53,54,55,56,57,58,59,60,61,62,63,64,65,66,67,68,69,70,71,72,73,74,75,76,77,78,79,80,81,82,83,84,85,86,87,88,89,90,91,92,93,94,95,96,97,98,99,100,101,102,103,104,105,106,107,108,109,110,111,112,113,114,115,116,117,118].

Many bacteria, fungi, and yeasts have been reported to be highly effective in OTA binding and degradation. Some strains of *Stenotrophomonas nitritreducens*, *Sphingomonas paucimobilis*, and *S. asaccharolytica* [70]; *Bacillus amyloliquefaciens* [67]; *Pseudomonas aeruginosa, Stenotrophomonas* sp., *Silanimonas* sp., *Luteimonas* sp., and *Lysobacter* sp. [39] have been found to be useful for OTA degradation. Some actinobacteria are also effective in OTA degradation and adsorption or can suppress the biosynthesis of OTA. For example, some *Streptomyces sp.* can degrade (approximately 20–50%) or adsorb (nearly 16–30%) OTA [56], whereas other *Streptomyces sp.* can suppress the gene expression responsible for OTA production by *A*. *carbonarius* [56]. *Phaffia rhodozyma sp.* can degrade nearly 90% of OTA within 15 days and adsorb nearly 23% of the same mycotoxin for 2 h [79]. According to some authors, the adsorption potential of microorganisms towards mycotoxins is dependent on some compounds in the cell wall, such as mannans or β-glucans [119], mannoproteins [120], and glucogalactans [58], but the culture conditions can also influence the adsorption capacity [121]. Different status of microorganisms (viable or dead) can also influence mycotoxin adsorption [59,79,85,101,122].

Some filamentous fungi such as *A. Japonicus* sp., *A*. *niger* sp., *A*. *carbonarius* sp. [97], *A. ochraceus*, *A. versicolor*, *A. fumigatus* sp., *A. Clavatus* sp., *A. Wentii* sp., *Cladosporium* sp., *P*. *spinulosum* sp., and *P. aurantiogriseum* sp. are also capable of degrading OTA [100,101,102]. In addition, various yeast strains such as *Yarrowia lipolytica* have been found to successfully degrade OTA [80,91].

Anaerobic *Eubacterium biforme* MM11 from the natural microbiota of swine intestine was also found to have a great capacity to degrade nearly 80–100% of OTA or AFB1 in the target liquid medium or corn substrate for nearly 24 h, and therefore could be used for the elaboration of some feed supplements [71].

OTA degradation is mainly realized by hydrolytic enzymes such as carboxypeptidase A [39,108]. However, OTA can also be degraded via the hydrolysis of its lactone ring [123], but the final compound in such a degradation possesses high toxicity, which is similar to that of OTA, as studied in rats [124,125].

The reported detoxification of AFs using *Lactobacillus* strains, such as *Lactobacillus rhamnosus*, was recently found to be attributed mainly to the binding of AFM1 or AFB1 [46,47]. Similarly, AFB1 detoxification in vitro by the probiotic yeast *Saccharomyces cerevisiae* was realized by the same mechanism as that of *Lactobacillus* strains (AFB1 binding) [82]. *Saccharomyces cerevisiae* bacteria were found to be very successful in AFB1 binding, whereas other strains such as *Phoma* sp., *Mucor* sp., *Rhizopus* spp. 663, 668, and 710, *Trichoderma* sp. 639, *Trichoderma harzianum*, some *Sporotrichum* strains, and *Alternaria* sp. were found to have the ability to destroy more than 65% of AFB1 content [92,93,94,95], and the *Flavobacterium aurantiacum* strain was found to be very successful in AFB1 removal [57].

DON was found to be transformed by *Eubacterium* strain BBSH 797 into the non-toxic metabolite de-epoxy-deoxynivalenol [61]. Similarly, ZEA and OTA have been reported to be detoxified by the yeast species *Trichosporon mycotoxinivorans* [73] via OTA cleavage into the less toxic OTα and phenylalanine [70] and ZEA transformation into non-toxic ZOM-1 [74]. In subsequent in vivo experiments, the same strains, *Eubacterium* BBSH 797 and *T. mycotoxinivorans*, were also found to detoxify the same mycotoxins [76,126]. The *T. mycotoxinivorans* strain was also found to meet all food safety requirements for feed supplements in the EU [75]. The commercial product Mycofix^®^ Plus^MTVINSIDE^ was created by Biomin GmbH (Austria) based on the potent OTA detoxification potential of *T. mycotoxinivorans* (MTV, 115). Subsequent experimental studies showed a strong protective effect of the same commercial product against OTA-provoked toxic effects in chicks, including poor feed conversion ratio, decreased body weight gain, and increased enzyme activity of serum aspartate aminotransferase (AST), lactate dehydrogenase (LDH), and γ-glutamyltranspeptidase, as well as against OTA-provoked pathological damage in the liver, kidney, bursa of Fabricius, and spleen [127].

Similarly, carboxylesterase was found to destroy FUMs in the porcine intestine, as the genetic code of this enzyme was previously isolated from a soil-dwelling microorganism and subsequently cloned into *Pichia pastoris* (recently renamed *Komagataella pastoris*) [77].

Various microbial agents with potent adsorption capacity for OTA also have great potential for industrial applications in food [84,128,129]. A good example in this direction revealed that within a 90-day fermentation process, the OTA content of 4 μg/mL can be decreased by 90%, 85%, and 73% in the must of red wine, rose, or white wine, respectively, due to adsorption by *S. cerevisiae* [128]. Similarly, *Candida intermedia 253* yeast cells included in calcium alginate magnetic beads were reported to be very effective in OTA adsorption from commercial grape juice, as more than 80% of OTA content (0.02 μg/g) was found to be adsorbed within an incubation period of approximately 48 h [84].

The yeast strain *Kluyveromyces marxianus* C2, isolated from porcine feces, has also been reported to decrease by more than 80% of OTA content in a YPD medium or in moldy corn [39].

An experimental study demonstrated that the PAT content in juice could be significantly decreased by up to 88% via the application of inactivated *Alicyclobacillus* spp. at a concentration of approximately 50 g/L [55]. PAT can also be degraded by *Byssochlamys nivea* (FF1-2) strain [96]. An in vitro study also demonstrated that *Rhodosporidium kratochvilovae* strain LS11 and *Sporobolomyces* sp. strain IAM 13481 are not susceptible to PAT toxicity and can degrade it into less toxic compounds such as desoxypatulinic acid and ascladiol [89].

#### Mechanisms of Protection and Industrial Applicability

Generally, the enzymatic degradation of mycotoxins has not been reported to be very useful in regard to AFs, FUMs, DON, and ZEA. In this regard, the reported detoxification of AFs was found to be attributed mainly to the binding process [46,47].

Some licensed probiotics containing *S. cerevisiae* LOCK 0140, *L. brevis* LOCK 0944, *L. plantarum* LOCK 0945, *L. paracasei* LOCK 0920, and *Yucca schidigera* extracts are other practical ways to decrease OTA levels in broiler feed, as reported by the decrease of 5 ppm and 1 ppm OTA levels by 55% and 73%, respectively, during fermentation within 6 h with the same probiotics [130].

Similar biological methods involving bioactive agents have been developed to decrease PAT and/or *Alternaria* mycotoxins in target fruits and their derivative products. These methods are based on fermentation or adsorption by yeast (incl. *Saccharomyces cerevisiae*) and/or enzymatic degradation. These methods often involve lactic acid bacteria (LAB), *Alicyclobacillus* spp., and other target bacteria [48,54,55,83,131]. The same bioactive agents do not usually provoke unwanted changes in product quality, but further investigation is required to clarify some safety issues and the intimate mechanism involved in such detoxification (including the optimal parameters) in order to fully develop such methods for successful application in the juice or fruit industry.

Several mechanisms of bioactive destruction or removal of PAT have been described, including biosorption by bacteria or yeast [132,133], destruction by enzymes [88], and the destruction of its toxic potential [134,135]. On the other hand, the presence of PAT in the contaminated substrate can cause the production of PAT-destroying enzymes by yeast, which is resistant to PAT [88,106,136,137]. For example, the yeasts *Saccharomyces cerevisiae* and *Gluconobacter oxydans* can degrade PAT into Z-ascladiol and E-ascladiol [60,83], *L. plantarum* can degrade PAT to hydroascladiol [48], whereas *Rhodosporidium paludigenum* can degrade PAT to desoxypatulinic acid, which is a less toxic compound [88].

Enzymatic degradation of PAT is the most important mechanism of detoxification in juices prepared from pome fruit [89], as some enzymes with antioxidative properties are involved in the removal of reactive oxygen species [138]. In this regard, target enzymes such as peroxidase and polyphenol oxidase have been reported to decrease PAT content in fruits [118]. For example, polyphenol oxidase, which is extracted from apples, has been reported to strongly decrease PAT content in apple juice. Such enzymes, like peroxidase and/or glucose oxidase, were found to reduce *Alternaria* mycotoxins not only in fruits but also in tomatoes. For example, the extract from horseradish peroxidase was reported to reduce the level of *Alternaria* mycotoxin alternariol (AOH) in tomatoes [117], glucose oxidase produced by *Aspergillus niger* was reported to reduce the AOH content in apples, and CotA laccase produced by *Bacillus licheniformis* ZOM-1 was reported to destroy AFs, ZEA, or AOH [116].

Enzymatic degradation has also been applied to OTA, and some proteolytic enzymes, such as carboxypeptidase A, trypsin, and α-chymotrypsin, were found to be successful in OTA hydrolysis as early as 1969; however, carboxypeptidase A was reported to be more successful in OTA hydrolysis [111]. The main mechanism of OTA degradation includes hydrolysis of its amide bond via hydrolytic enzymes such as carboxypeptidase A, lipase A, protease A, and ochratoxinase [39,108].

Among the commercially purified enzymes, the crude enzyme Ancex is reported to be very powerful in OTA degradation when compared to other enzymes, such as Pancreatin, Protease A, or Prolyve PAC [109]. The crude metalloenzyme produced by *Aspergillus niger* was also reported to be very powerful in OTA hydrolyzation compared to carboxypeptidase A [115]. Purified recombinant ochratoxinase has been reported to be more effective in OTA hydrolysis than carboxypeptidase A at the target pH and temperature [139]. Other enzymes produced by *Aspergillus tubingensis* (M074 and M036) have also been reported to be very effective for OTA removal (>90% of OTA was removed at 25 °C and pH 5) [99].

A good example of industrial application is the commercial product Mycofix^®^ Plus^MTVINSIDE^ elaborated by Biomin GmbH (Austria), based on the good OTA detoxification potential of *T. mycotoxinivorans* (MTV, 115).

Obviously, a lot of microorganisms or fungi/yeast, having powerful OTA-adsorbing or degrading capabilities, in addition to the target enzymes cloned or produced by such fungi/yeast/microorganisms or by the pancreas of some animals, could receive or already have received great application prospects in the food/feed industry.

The high efficacy and absence of pollution in processed feeds, fruits, and derived products could explain the leading role of biodegradation as a promising new strategy for mycotoxin control. Nowadays, it is discovered that many microorganisms or fungi have the potential to destroy or decrease mycotoxin content in feeds/foods and their derived products. In this regard, further efforts are required to clarify the mechanisms responsible for the detoxification process and isolate the enzymes involved.

### 2.2. Antagonistic Microorganisms, Fungi, or Yeast with Fungicidal Properties Against Mycotoxin Contamination

A practical and safe biological method for preventing mycotoxin contamination of feeds/foods and their derived products is treatment with some microbial/fungal antagonists, which appears to be a powerful alternative to some conventional fungicides. The development of safe biological technologies to inhibit the growth of toxinogenic fungi is highly desirable. For example, *Bacillus subtilis* was found to prevent the growth of *Fusarium* fungi [34].

Atoxigenic *Aspergillus flavus* strains were reported to decrease AFs levels in treated feeds/foods and derived products [140] because they develop in the same ecological space/niche as mycotoxin-producing fungi and displace them. For example, spores of atoxigenic *A. flavus* strains inoculated on sorghum or barley could be a safe and practical technology for preventing feed contamination with AFs. Such inoculation can completely displace AF-producing strains and could be a safe and practical strategy for reducing the preharvest AFs content in crops [141].

Therefore, the use of naturally encountered atoxigenic *Aspergillus* strains for competitive exclusion of toxigenic strains appears to be a powerful method for controlling AFs content in foods/feeds. This strategy of using atoxigenic *Aspergillus* strains against toxigenic strains could be applied to control AFs production in the field, with a subsequent decrease in AFs contamination during storage.

This biocontrol approach against AFs contamination was first introduced in 1993 [142] and subsequently applied in many other countries worldwide [143]. However, such a strategy requires a predominance of atoxigenic strains above toxigenic ones in the field circumstances [144]. This could be achieved by applying the same atoxigenic strains, also known as biopesticides, in the field to ensure competition with toxigenic strains and the suppression of their multiplication, resulting in a decrease in AFs production [145] (Table 2) [34,56,90,131,135,140,141,145,146,147,148,149,150,151,152,153,154,155,156,157,158,159,160,161,162,163,164,165,166,167,168,169,170,171,172,173,174,175,176,177,178,179,180,181,182,183,184,185,186,187,188,189,190,191,192,193,194,195,196,197,198,199,200,201,202,203,204,205].

*A. flavus* strain NRRL21882 and *A. parasiticus* strain NRRL21369 were also reported to be very effective biocontrol agents against AFs contamination in peanuts when applied under field conditions at preharvest time or at postharvest storage. Another atoxigenic strain, BN30, was found to be effective in preventing AFs contamination in maize in Africa [184]. In Australia, non-toxigenic strains have been reported to reduce AFs contamination in peanuts by up to 95% [182]. In China, approximately 30 non-toxigenic *A. flavus* strains were examined as biocontrol agents, and the AF051 strain was reported to be most effective in the control of AFs, with up to a 99% decrease in peanut fields [185].

A decrease in the AFs contamination of maize by up to 65–94% was achieved in a four-year study using atoxigenic CT3 and K49 strains [145]. Similarly, a potent decrease in AFs has been reported in groundnut fields using the atoxigenic AR100G, AR27, and AFCHG2 strains of *A. flavus* in Argentina [186]. *Aspergillus niger* strain FS10 has also been reported to decrease AFs production in the field [187,188].

*Penicillium chrysogenum* strain RP42C was also reported to suppress the growth of toxigenic *Aspergillus* strains [189].

Some yeast strains, such as *Kluyveromyces* spp., *Debaryomyces hansenii* strain BCS003, *Candida maltose, Pichia anomala, Saccharomyces cerevisiae* RC016, and *Saccharomyces cerevisiae* RC008, have been found to significantly decrease the growth of *Aspergillus* spp. and subsequent AFs production [147].

*Trichoderma* spp., *T. viridae, T. harzianum, T. Auroviride*, and *T. longibrachiatum* have been reported to be very effective against AFs production in the field at rates between 50% and 80% [147,190,206]. *Trichoderma* spp. were also found to decrease AFs contamination in sweet corn and groundnuts by 65% and 57%, respectively [157].

Some bacterial species, such as *Lactobacilli, Streptomyces, Ralstonia, Stenotrophomonas, Pseudomonas, Burkholderia*, and *Bacillus* have been reported to be effective against AFs contamination. *Bacillus* spp., such as *B. subtilis, B. megaterium, B. mojavensis, B. amyloliquefaciens*, *B. mycoides, B. pumilus, B. cereus*, and *B. mojavensis* were found to be effective biocontrol agents against AFs contamination [147,148,149,150,151]. *Bacillus megaterium* was reported to prevent nearly 100% of AFs production in a broth medium [148], whereas *Bacillus subtilis* was found to control the development of *Aspergillus parasiticus* (nearly 92%) and subsequent AFs production by up to 100% [149].

*Pseudomonas chlororaphis* strains isolated from maize have been reported to inhibit *A. flavus* development by nearly 100% [152]. *Pseudomonas fluorescens* was found to suppress the conidial germination of *A. flavus* by nearly 20% [178], in addition to inhibiting AFB1 production (> 99%) in peanut medium [177]. *Pseudomonas protegens* strain AS15, isolated from rice grains, was found to suppress up to 83% of AFs production, in addition to suppressing the development of *A. flavus* (up to 68%) [179].

*Lactobacillus* (LAB), such as *L. delbrueckii, L. plantarum, L. reuteri, L. acidophilus, L. rhamnosus, L. paraplantarum, L. fermentum, L. Casei*, and *L. pentosus*, have also been reported to be effective against AFs; however, *L. plantarum* was found to be the most effective against AFs production [173,174,175].

*Lactobacillus plantarum* B4496, *Lactobacillus brevis* 207, and *Lactobacillus sanfranciscensis* BB12 isolated from fermenting cocoa were found to have good in vitro antifungal activities against the three OTA-producing fungi, *A. niger*, *A. Carbonarius*, and *A. ochraceus*, with suppression capabilities ranging from 15% to 67% [176].

*Streptomyces* strains, such as *S. anulatus, S. yanglinensis, S. roseolus*, and *S. alboflavus*, were also found to be highly effective against aflatoxigenic fungi, such as *Aspergillus flavus* [153,154,155].

The bacterium *Serratia marcescens* strain JPP1, isolated from peanut shells, was found to suppress AFs production by nearly 98% and the subsequent growth of *A. parasiticus* by nearly 95% [180]. *Nannocystis exedens* was also found to significantly suppress the growth of *A. flavus* and *A. parasiticus* [156].

In vitro antifungal activities against OTA- and AFs-producing fungi were found in fermented cell-free supernatants of *Paenibacillus chibensis* CECT 375, *Bacillus amyloliquefaciens* CECT 493, and *Pantoea agglomerans* CECT 850, owing to high levels of acetic acid, lactic acid, phenyllactic acid, and benzoic acid [172].

*Rhodotorula glutinis* yeast strain LS11 was found to reduce the PAT content and destroy mycotoxins in vitro [90]. It has also been reported that the initial PAT content could be reduced by approximately 80% after 2 days of incubation with the antagonistic yeast *Pichia ohmeri*, and 15 days later, PAT was completely undetectable [191].

Incubation with *Pichia caribbica* yeast for 15 days has also been found to reduce PAT levels in apples [192]. A decrease in PAT levels was reported after inoculation with other biocontrol agents, such as *Pantoea agglomerans CPA-1* and *Candida sake CPA-2* [193], or some ascomycota yeast species, such as *Candida guilliermondii P3* and *Pichia ohmeri 158* [181]. The reduction in PAT levels was thought to be a result of fruit protection against infestation by the PAT-producing *P. expansum* strain and/or PAT absorption, but not metabolization [207].

Other antagonistic yeasts/microorganisms or their extracts, which can suppress fungal growth and production of PAT, have been reported mainly for apples and include some laboratory cultures, such as *Torulaspora delbrueckii* [195] and *Candida membranifaciens* [194], in combination with silicon. The decrease in PAT production by *P. expansum,* when co-incubated with antagonistic yeast was attributed to the growth inhibition of *P. expansum* and subsequent decrease in its mycotoxin production [137]. Some LAB strains and cell-free LAB supernatants were also found to suppress the growth of *P. expansum* and *Aspergillus parasiticus* by 58% and 73%, respectively, in a liquid medium after 48 h of incubation [135].

Other microorganisms, such as *Bacillus subtilis* and *Pseudomonas fluorescens*, have been shown to decrease PAT content in apples [171]. Potent control of the development of fungal strains *P. expansum* and *Botrytis cinerea* was also achieved by inoculation with some *Pseudomonas syringae* isolates after nearly a month of storage [131].

The reported inhibition by such bioactive agents could be attributed to competition for available space and/or nutrients, the synthesis of target bioactive antagonistic compounds, which inhibit spore germination and fungal growth, and/or direct predation by antagonist rivals [170,196].

#### Mechanisms of Protection, Factors Influencing Antifungal Activity and Industrial Applicability

The modes of inhibitory effects of the protectors include the following: (1) competition for nutrient and living space among antagonists, which grow rapidly and occupy ecological niche of pathogens and thus displacing them; (2) inhibition of fungal growth and reduction of target fungal infection and colonization; (3) inhibition of mycotoxin production. The inhibitory compounds are usually secondary metabolites and antimicrobial substances such as lipopeptides, protease antibiotics, bacteriocins, enzymes, and organic acids, such as aflastatin A or dioctatin A, etc., which suppress mycotoxin production [162,163,164].

The main mechanisms of inhibitory actions include the following: (1) destabilization or destroying cell wall structure; (2) affecting the activities of mitochondria, nuclei, or cellular membranes; (3) downregulation of expression of mycotoxin synthesis-related genes or a combination of several of the above-mentioned mechanisms [147,162,163,164].

The Bacillus spp. usually produced a wide range of antimicrobial compounds, grow rapidly, and are safe species; therefore, they have often been investigated as biological agents [147,148,149,150,151]. Bacterial antagonists were reported to be dominant (61%) compared with antagonistic fungi (27%) or yeast (12%), but currently, the introduction of non-toxigenic *A. flavus* into the field appears to be the most promising strategy for the prevention of preharvest AFs contamination [147], and *A. flavus* strain NRRL21882 is commercially available as a biopesticide Afla-guard^®^ [183]. Currently, only non-toxigenic *A. flavus* strain NRRL21882 and NRRL18543 have been commercialized in terms of application in the field. The same biopesticide and biocontrol approach proved to be effective on peanuts [158], cottonseed [159], and corn [160]. The application of non-toxigenic *A. parasiticus* in the field was also found to reduce AFs contamination during storage time [161]. However, *Trichoderma* species also show a good potential to be commercialized in the future [157].

Usually, a strong relationship is observed between the inoculum rate and effectiveness of most biocontrol agents. Soil temperature also influences the effectiveness of biocontrol agents and should be above 20 °C in most cases. Therefore, late spring is the optimal time for the application of atoxigenic biocontrol agents. The first biocontrol agent (strain *Aspergillus flavus* AF36) against AFs contamination of cottonseed was registered in Arizona, USA, which was subsequently found to be effective against toxigenic *A. flavus* strains in corn, with a 70–90% decrease in AFs production in cotton and peanut reported in some field experiments when applying such atoxigenic *Aspergillus* strains [146].

Similarly, the inoculation of biomodulating microorganisms that are capable of suppressing *P. expansum* growth or destroying its metabolism could be a practical and safe technology for decreasing PAT content during the storage of some fruits. Postharvest decay induced by *P. expansum* can be effectively prevented using such a powerful and safe strategy for suppressing PAT contamination in stored fruits [90,191]. In this regard, dipping treatment of two apple cultivars in a *Pseudomonas fluorescens* cell suspension prior to dipping treatment in a *P. expansum* spore suspension was shown to inhibit fungal development on apples at postharvest time during commercial storage, which was reported to be comparable to that of commercial fungicides [170].

Mycotoxin production by fungi is usually influenced by temperature, water activity, pH values, nutritional source, among others. The antifungal activity of various biocontrol microorganisms, fungi, or yeast is also influenced by the same or similar factors and various environmental conditions. For example, the bioactivity of *L. plantarum*, which is one of the best protectors against AFs-producing strains, increases in low pH values, similar to some fungi or yeast species [165]. The best antagonistic activities of yeast strains *Saccharomyces cerevisiae* RC008 and RC016 are at pH value 4 [166]. However, the best pH value for each antagonistic fungus, microorganism, or yeast is species dependent.

Temperature and water activity are also of high importance for the antifungal efficiency of antagonists. For example, the yeast strain *Debaryomyces hansenii* can stimulate the AFs production of *A. parasiticus* at water activity of 0.99 but significantly reduce AFs production at water activity of 0.92 [167].

In regard to temperature, it is reported that the maximum activity of protein-degrading enzyme protease P6281, produced by the fungus *T. harzianum*, towards AFs is 40 °C [168], whereas some other antifungal species have different optimal ranges of antifungal activity and mycotoxin production.

Similarly, incubation time is also of significant importance, but the best inhibitory activity is usually anticipated after an incubation period of 3–4 days [169].

Obviously, water activity, pH value, incubation temperature, and nutritional source are of great importance for the growth of fungal species or yeasts and their antifungal activity. In this regard, profound practical work is required to identify the effective biocontrol agents before their application and commercialization.

## 3. Natural Herbal Supplements Having Powerful Protection Against Toxicity of Mycotoxins

### 3.1. Plants and Herbal Supplements with Powerful Protective Properties Against Target Mycotoxins

A powerful way to protect against the harmful toxic properties of mycotoxins on animal and human health is the addition of various natural mycotoxin-detoxifying supplements, such as herbs or plants that possess protective or antidote properties against mycotoxins [6,17,208,209,210,211,212,213,214,215,216]. For example, more than 7000 species of plants in India are currently used for medical purposes to cure various diseases or ailments [217] (Table 3) [9,10,11,14,15,17,208,209,210,211,212,213,215,216,218,219,220,221,222,223,224,225,226,227,228,229,230,231,232,233,234,235,236,237,238,239,240,241,242,243,244,245,246,247,248,249,250,251,252,253,254,255,256,257,258,259,260,261,262,263,264,265,266,267,268,269,270,271,272,273,274,275].

Plant extracts or herbal supplements provided to the feedstuffs were found to protect against OTA-provoked decreases in weight gain among stock chicks [17,209,210] and prevent OTA-provoked decreases in egg production among laying poults [6]. Such protection against the toxic effects of OTA and improvement in OTA elimination from the organism was observed about 5% total water extract of dried leaves of artichoke (*Cynara scolymus L*) prepared as a steam infusion and administered to chicks at 5 mL/kg. b.w. via drinking water or forage [17,208,209,210]. The increase in the hepatobiliary route of OTA excretion via enhanced biliary secretion was attributed to the cynarine content in the artichoke extract [210], and the urinary route of OTA excretion was also improved by the increase in diuresis among artichoke-treated chicks [17,210]. Artichoke extract was also found to decrease OTA content in the kidneys and liver due to its increased elimination [209]. The edematous changes in OTA-treated chicks were also decreased due to vasoconstrictive and permeability-decreasing effects of the same herb [17,210], whereas hepatoprotective properties against OTA-provoked liver damage were attributed to the high content of flavonoids and cynarin in the artichoke extract. The improvement in diuresis by the same additive contributed to the decrease in serum glucose level, which was increased by OTA treatment [17,210].

Rosallsat, which is a commercial plant extract of bulbus *Allii Sativi* and seminum *Rosae caninae*, was also reported to have protective properties against OTA when taken at a dose of 0.6 mL/kg b.w. per day as a supplement to the chicken feedstuffs [210]. The bioactive compound “allicin” and the large quantity of some vitamins (e.g., E, A, F) in this plant extract were supposed to protect against OTA toxicity. Suppression of lipid peroxidation by Rosallsat was suggested to ameliorate the OTA-provoked increase in lipid peroxidation [276], which is responsible for cell membrane damage, subsequent influx of cellular Ca, and impairment of cellular metabolism and necrosis [277].

Roxazyme-G, a polyenzyme complement produced by the fungus “*Trichoderma*”, was found to be another powerful protector against OTA toxicity when administered at 200 ppm in chicken feedstuffs [209]. The improvement in energy metabolism by this supplement was responsible for the protective effect against OTA-induced disturbances in energy metabolism and the subsequent decrease in egg production. This assumption was supported by the decreased levels of serum glucose in chicks [209] and increased egg production in laying hens supplemented with the same polyenzyme complement [6]. The authors suggested that such natural feed additives could be used as a practical approach for the safe utilization of OTA-containing forages for chicks, thereby preventing the scrapping of such forages [209].

Ground sesame seeds have been reported to be another powerful natural protector against the OTA-provoked immunosuppression of humoral immune response and impairments in differential WBC count when administered at 80000 ppm to chicken feed. The same protection was attributed to the improved protein synthesis and enhanced division of immune cells, which are disturbed by OTA, and is due to the high level of phenylalanine in sesame seeds, which is a structural analog of OTA with antidote effects against this mycotoxin [209]. However, large-scale use of sesame seeds is impractical because of the high cost of such protection.

Protective effects of the herbal supplements *Silybum marianum*, *Withania somnifera*, and *Centella asiatica*, administered at feed levels of 1100, 4000, and 4600 ppm, respectively, were found against the immunosuppressive and toxic properties of OTA in broilers vaccinated against Newcastle disease and treated with 5 ppm OTA via the feeds [211]. The most powerful protective effects of *W. somnifera* and *S. marianum* were reported against OTA-provoked immunosuppression and damage to the kidneys and liver; however, the nephroprotective effect was stronger in poults supplemented with *S. marianum*, as evidenced by changes in biochemical and pathomorphological findings and relative organ weights. The use of these herbs has been suggested as a practical approach to combat the deleterious effects of OTA and to safely utilize OTA-contaminated forage. The mechanism of protection of these herbs was found to be different: *S. marianum* and *W. somnifera* were defined as good stimulators and protectors of the immune system; *S. marianum* was reported to be a good protector against OTA-induced damage in the liver and kidneys; and *W. somnifera* was reported to protect mainly against the hepatotoxic effects of OTA [211].

The hepatoprotective properties of *S*. *marianum* were also reported against liver damage induced by AFs, as evidenced by the decreased activities of the enzymes AST, alanine aminotransferase (ALT), and alkaline phosphatase (ALP) in the serum of AFs-compromised poults [219]. Such protection has been reported for Silymarin or Milk thistle (a seed extract of *S. marianum*) with regard to AFs-provoked liver damage in poults [220]. Body weight gain was significantly improved in poults supplemented with *S. marianum* or silymarin and exposed to AFs [211,219,220]. In addition to the improved gain in body weight, the feed conversion ratio was also improved in response to such treatment, and the same parameters were similar to those in poults treated with a toxin binder [219]. A similar increase in body weight gain was observed in rats protected with *W. somnifera* [235]. Supplementation of animal/chicken forages with either of the two herbs was found to significantly improve feed utilization.

Protection by milk thistle has also been reported against FB1-provoked liver or kidney damage in rats [278].

Potent protection by silymarin was also observed in AF-induced diabetic nephropathy [221] or cisplatin-induced kidney damage in rats [223] or against gentamicin-provoked damage in the kidneys of dogs [222].

The protective properties of silymarin against OTA-provoked damages to internal organs and the deleterious effects on biochemical indices, such as increased levels in uric acid, serum glucose, and enzyme activities of ALT and AST in OTA-compromised chicks, confirmed its powerful protection of the liver and kidneys [213].

A dose-dependent protective effect of silymarin against OTA-provoked immunosuppression was observed in another experiment. Silymarin and/or Vitamin E administered alone or together were reported to be powerful protectors of the immune system, but mainly at OTA exposure levels below 2 ppm [218].

A powerful liver-protective effect of *S. marianum* or silymarin was also observed against some other kinds of experimental damages in the liver of rats, as evidenced by the decrease in ALT, AST, ALP, lipid peroxidation, and tumor necrosis factor [225,226,227,228].

Silymarin and *S. marianum* are known to possess powerful antioxidative properties in addition to their strong immunomodulatory, membrane-stabilizing, hepatoprotective, and nephroprotective properties [211,213,219,220,227,228,231,279,280,281,282,283].

The protective properties of *S. marianum,* silymarin, or *W. somnifera* against mycotoxin-induced suppression of humoral or cell-mediated immune responses have been confirmed in other studies on the immunomodulatory properties of these herbs [231,233].

The suggested protective mechanism of *S*. *marianum* and silymarin was attributed to the suppression of lipid peroxidation and increased levels of endogenous antioxidants, which provided integrity to cellular membranes and prevented the leakage of some enzymes responsible for cellular death [227,229,230]. These antioxidant properties of silymarin or *S. marianum* are attributed to the powerful suppression of free radical production in the metabolism of toxic substances, increased levels of hepatic glutathione, and increased antioxidant defense in the liver [284]. The powerful protective effect of silymarin is due to some flavonoids, such as silybin, which possess powerful biological activities, including strong nephroprotective and hepatoprotective effects [279,280,285].

The herbal supplement *W. somnifera* has been reported to have similar immunoprotective and antioxidative effects [233], to suppress lipid peroxidation, to protect against liver damage [232], and to have potent neuroprotective properties [211,234]. This protection has been attributed to bioactive substances, such as saponins, steroidal lactones, and alkaloids [235].

It seems that the herbal supplements *W. somnifera*, silymarin, or *S. marianum* can be used as supplements to forage, with or without mycotoxin binders, to ensure the possible amelioration of the harmful effects of mycotoxin-contaminated feedstuffs in poultry farms [219,220]. Obviously, the same herbal supplements could provide a safe approach for the utilization of mycotoxin-containing feedstuffs, minimizing possible losses from reduced body weight of the birds, other possible losses from health issues, or discarding such mycotoxin-contaminated feed [1].

A protective effect on the gastric and intestinal mucosa and/or intima of vessels has been reported for the herbal additive *C*. *asiatica* [236,237], providing ameliorating effects against toxic damages caused by OTA or DON on the gastrointestinal mucosa and permeability of vessels [211]. This herb is recommended as a protective agent against oxidative stress and the deleterious effects of free radicals on mucosal integrity, thereby improving its barrier and defensive capabilities [236,237], which can be damaged by these mycotoxins [1]. The protective capability of the same herb against OTA-provoked immunosuppression and liver or kidney damages was slightly expressed [211,241].

A polyherbal feed supplement (“Growell”) was also found to be a good protector in some experimental cases of aflatoxicosis, ochratoxicosis, or combined mycotoxin intoxication in poults [262,263].

A protective effect of another herb, *Tinospora cordifolia* was found against OTA-provoked biochemical and oxidative changes in the spleen of mice [215,216]. Considering that many mycotoxins can induce oxidative stress [216,286] and worsen animal and human health, the antioxidant effects of herbs are highly appreciated. The protective capability of the *T. cordifolia* extract was attributed to its powerful antioxidant potential against oxidative stress and, therefore, it could protect against many mycotoxicoses [215,216]. The antioxidant properties of this herbal supplement were found to be related to its powerful radical scavenging potential against reactive nitrogen species (RNS) and reactive oxygen species (ROS) [287]. It is well known that ROS and RNS usually increase under the action of many mycotoxins, such as OTA [215,216]. The scavenging properties against ROS and RNS are related to the tannins and phenolic compounds in these extracts [288]. The same extract was also reported to ameliorate the genotoxicity of OTA, incl. 8-OHdG (8-hydroxy-2′-deoxyguanosine) genotoxic biomarker [215,216].

*Glycyrrhiza glabra* (Liquorice) is also a common herb often applied to various human ailments in the East and West [261] due to its potent hepatoprotective and antioxidative action [255,259,289] as well as its strong immunostimulating properties [212,258]. This protective effect is attributed to some biologically active compounds, including saponin glycyrrhizin, flavonoids, hispaglarbidin B, glabridin, licocoumarin, isoliquiritigenin, and some others [257], which are responsible for many natural protective properties of this herb, such as its antiviral, antibacterial, anti-inflammatory, hepatoprotective, cardiotonic, expectorant, antidiabetic, and antithrombotic effects [224,261]. That is why *Gl. glabra* could protect against the immunosuppressive, hepatotoxic, and pulmonary toxic effects of mycotoxins such as AFs, OTA, DON, and FUMs [212].

*T. cordifolia* has also been reported to have anti-inflammatory, immunostimulating, hepatoprotective, diuretic, antidiabetic, and anti-neoplastic properties, and to suppress lipid peroxidation [238,244,252,290], all of which are responsible for its protective effects against the nephrotoxic, hepatotoxic, immunosuppressive, and carcinogenic effects of some mycotoxins, improving mycotoxin elimination via the kidneys. This herb has been used to treat health ailments, such as dysentery and urinary diseases [290], which have often been reported in animals with mycotoxicoses.

Powerful protective properties against the suppressive effect of OTA on body weight gain and the associated pathomorphological and biochemical changes have been reported for the *Gl. glabra* and *T. cordifolia* herbs added to the feedstuffs for poults at 6600 and 4000 ppm, respectively [212]. The OTA-provoked decrease in relative organ weights, body weight, and antibody titer in chicks immunized against Newcastle disease was less expressed in poults supplemented with *Gl. glabra* or *T. cordifolia* in addition to OTA treatment, compared to poults treated with only OTA. The protective effect of both herbs on the immune system was supported by the higher relative weight of lymphoid organs of OTA-treated poults supplemented with the herbs as compared to poults treated with OTA only. The protection of both herbs against OTA-induced liver damages was also observed and better expressed in poults supplemented with *Gl. Glabra,* being associated with milder pathological damages and lower serum AST levels. *T. cordifolia* has also been reported to have good protective properties in the bone marrow and kidneys of poults, as supported by the lower serum levels of uric acid compared to poults without protection [212].

*T. cordifolia* [242] and *Gl. glabra* [258] extracts were also found to improve antibody production in vivo. *T. cordifolia* can improve phagocytic activity without significantly influencing humoral or cell-mediated immunity [245,291]. It is important to mention that *T. cordifolia* was found to influence production of cytokines and stimulate the immune system by stimulating differentiation of B cells and activation of T cells [239,243].

*T. cordifolia* extract was also found to inhibit α-glucosidase, which is probably responsible for its antidiabetic properties [253,254] and ability to decrease serum glucose levels [288]. Therefore, this herb was reported to decrease the levels of serum glucose in poults that ingested OTA via their diet and additionally supplemented with *T. cordifolia* [212], thus ameliorating OTA-provoked increases in serum glucose levels.

The powerful hepatoprotective potential of *T. cordifolia* has been reported against various experimental liver damages [248] induced by carbon tetrachloride [249,252], bile salts [245], and lead nitrate [250]. The same herb has been reported to suppress lipid peroxidation [240,246], ameliorating damage to internal organs due to OTA-induced increase in lipid peroxidation [212].

*T. cordifolia* was found to have protective effects against kidney and liver damages induced by AFs [247]. *T. cordifolia* was also reported to have protective effects on the gastrointestinal system [251], which could be attributed to the prevention of damages provoked by free radicals on the gastrointestinal mucosa [216], ameliorating the toxic effects of mycotoxins, such as DON or OTA [212] on the mucosa of the intestine.

It seems that both herbs *T. cordifolia* and *Gl. glabra* possess powerful antioxidative effects, as well as organ-protective and immunostimulating properties [246,256,257], and can be potent inhibitors of lipid peroxidation [260]. Therefore, both herbs could be potent protectors against the toxic effects of mycotoxins such as OTA, AFs, DON, or FUMs by decreasing lipid peroxidation and/or oxidative stress [216,292], liver and kidney damage [247], gastrointestinal damage [251], and immunosuppression [212].

The actual mechanism of protection of both herbs *Gl. glabra* and *T. cordifolia* in the previously mentioned experimental studies with chicks or rats could be partially explained by the suppression of lipid peroxidation and the increased levels of endogenous antioxidants that support the integrity of the cellular membrane and prevent possible leakage of target enzymes into the cytosol, which can lead to cellular death. On the other hand, the immunosuppressive effect of mycotoxins is often responsible for some neoplasms, because the important function of suppressed natural killer cells is to kill any neoplastic cells that are excluded from the physiological community [10,14,293]. Therefore, these herbs may also have anticarcinogenic properties. In addition, the same herbs were found to have a potent antibacterial or antiviral effect, thus simultaneously serving as powerful immune boosters [211] and possibly preventing secondary bacterial diseases induced by mycotoxin-provoked immunosuppression [18]. Moreover, together with the above-mentioned protective properties, these herbs have been found to protect against various kinds of toxic damage to the kidney, liver, and gastrointestinal system [216,224,248] and, therefore, could ameliorate the damage induced to the same organs by mycotoxins such as AFs, OTA, DON, or FUMs in animals or poultry [212].

A nephroprotective effect was also reported for oleanolic acid, which was found to ameliorate OTA-induced apoptotic damages and increase cell viability of epithelial cells from the proximal tubules of human kidneys (HK-2) [275]. The same natural compound is present in target medicinal plants and fruit skins.

Another bioactive compound possessing nephroprotective properties, which is present in some medicinal plants and cuticular waxes of some fruits, is ursolic acid. It was also reported to ameliorate mitochondria-mediated apoptosis in HK-2 cells induced by OTA treatment [273]. Pretreatment with 1 μM ursolic acid was also found to alleviate cell death and ROS production induced by OTA exposure in human embryonic kidney cells [274].

Turmeric powder, which is another natural product, was found to be a good alternative to mineral binders and has been reported to ameliorate AFB1-provoked toxic damage, such as increased lipid peroxidation in the livers of farm animals and poultry [265]. It was also found to increase the hepatic gene expression of target antioxidant enzymes (e.g., SOD2 and CAT) and decrease the contamination levels of AFB1 in the liver of broilers [265].

The methanolic extracts of *M. oleifera, A. leiocarpus, I. asarifolia, B. refescens*, and *G. senegalensis*, were also found to have potent antioxidative effects, which were attributed to the presence of flavonoids, alkaloids, and tannins. It has been reported that the DPPH free radical scavenging activities of methanolic extracts of *M. oleifera* and *A. leiocarpus* were the best among all examined extracts [264]. *Desmodium ramosissimum* methanolic extract has also been shown to have similar antioxidative properties and DPPH-free radical scavenging activity, and is widely used in traditional medicine [294].

Unfortunately, most reports on the antioxidative properties of plant extracts have not been studied in practice as possible protective agents against the deleterious effects of mycotoxins. It is worth mentioning that some methods of maize processing, such as treatment with lime water in the production of tortillas, can significantly reduce AFs content [36]. In addition, a synergistic interaction was observed in the destruction of AFB1 between lemon juice, citric acid, and heating/frying in pistachios contaminated with AFB1. Unfortunately, such processing can deteriorate some of the valuable taste qualities of the treated product [295].

#### Industrial Applicability and Limitations

By analyzing the available data in the literature, we can conclude that some herbs such as *T. cordifolia, W. somnifera, Gl. glabra*, silymarin, and *S. marianum*, or polyenzyme complements such as Roxazyme-G produced by the fungus “*Trichoderma*”, could be powerful protectors against mycotoxin toxicity, in addition to natural mycotoxin binders, and could ensure better utilization of mycotoxin-contaminated feedstuffs by improving the weight gain of mycotoxin-compromised commercial chicks/animals.

It seems that herbal or enzyme protections could be introduced in practice for large-scale use to ensure the safe utilization of such mycotoxin-contaminated feedstuffs [211,212]. The polarity of therapeutic compounds in herbs suggests the use of polar solvents for extraction [212].

The eventual economic loss resulting from the scrapping of mycotoxin-contaminated feedstuffs or the loss of body weight gain of animals/poults exposed to such feedstuffs could be avoided only by investing funds to purchase such herbal or enzyme products. In order to find suitable protectors for each mycotoxin, it is necessary to know in depth the specific mechanisms of its toxicity. Therefore, additional efforts are required to elaborate and implement such protection in large-scale applications.

However, it should be emphasized that there are some legislative restrictions on the use of food and feed additives in the EU. The restrictions for food additives include authorization and listing in the EU’s positive list, based on safety assessment and technological need. The Feed Additives Regulation (Regulation (EC) No. 1831/2003) of September 2003 establishes a common procedure for authorizing feed additives and lays down rules for their placing on the market, labeling, and use. In addition, EC Regulation No. 429/2008 of 25 April 2008 gives detailed rules for the implementation of EC Regulation No. 1831/2003 in regard to the preparation and presentation of applications, as well as the assessment and the authorization of feed additives.

### 3.2. Herbs and Plants Suppressing the Growth of Fungi and Production of Mycotoxins

Some bioactive compounds in herbs and plant extracts, such as flavonoids, polyphenols, silymarin, and carotenoids, have potent antifungal properties. The same compounds may suppress the growth of fungi such as *Aspergillus flavus*, preventing contamination of feedstuffs or food commodities with AFs [198,199,200,296,297] and, therefore, could be used in practice to prevent possible contamination with mycotoxins.

Plant extracts have also been found to suppress the growth of fungi that produce PAT and *Alternaria* mycotoxins. Some essential oils, such as clove and cinnamon oil, have been found to reduce PAT level in apples [201]. Garlic extract was found to be effective against *Alternaria* mycotoxins in tomatoes, whereas plant extracts of essential oils and/or monoterpenoids [203], as well as garlic extract and/or exposure to garlic vapor, strongly suppressed the fungal growth of *Fusarium oxysporum* and/or *P. expansum* in apples [205].

Natural extracts of orange peel and cistus were found to have good antifungal activity against the toxigenic *Aspergillus carbonarius* strain in a grape-based medium at concentrations of 10 and 20 mg/mL, whereas Eucalyptus extract was reported to reduce OTA production by up to 85% at a concentration of 10 mg/mL, with a slight influence on fungal growth [202].

Natural antioxidants have also been reported to be very effective in controlling postharvest fungi and suppressing PAT production [60], including fungal control and the production of mycotoxins such as AFs or OTA [204]. Other antioxidants such as vanillic acid have also been found to be useful in suppressing OTA production [197].

## 4. Some Natural Compounds or Vitamins Possessing Protective Effects Against Mycotoxicoses

Some energy boosters such as Roxazyme-G were reported to be effective against OTA-induced suppression of energy metabolism [209]. Some known suppressors of lipid peroxidation, such as Rosallsat [209,210], are also used as antidotes against OTA-provoked increases in lipid peroxidation, which is another important mechanism of OTA toxicity [276].

The protective properties of phenylalanine have also been reported against OTA-induced changes in blood biochemistry and pathology in rats. The number of OTA-induced neoplastic changes in rats supplemented with 20 ppm phenylalanine and exposed simultaneously to 10 ppm OTA was similar to that in rats exposed to two times lower OTA concentrations, which suggests a protective effect against the carcinogenic effect of OTA [14,15].

Protection of phenylalanine was also reported against OTA-induced teratogenic effects and malformations in mice supplemented with 20 ppm phenylalanine to the feeds [11], which confirmed its specific protective properties in this direction.

The reported protective properties of phenylalanine against OTA-provoked immunosuppression in humoral immune response were defined to be a result of increased protein synthesis, which is damaged by OTA, and to the consequent improvement of the division of immune cells, which is suppressed by OTA [209].

A slight protection of phenylalanine against OTA-provoked decrease in egg production was also reported in laying hens treated simultaneously with or without OTA and phenylalanine [6], suggesting a wide range of protective properties of the same antidote against the different toxic effects of OTA.

Ascorbic acid supplementation (300 ppm) in the diet of laying hens exposed to 3 ppm OTA was found to ameliorate egg production, including the number and weight of eggs [271,272], suggesting the protective properties of vitamins against the toxic effects of mycotoxins (Table 3).

Similar protective effects were reported for ascorbic acid and vitamin B, which have been found to facilitate PAT degradation [267]. Given that ascorbic acid and ascorbate are present naturally in many fruits, such as apples, and are additionally found to be capable to decrease PAT content in apple juice [268,269,270], their large-scale use at industrial levels is advisable. PAT degradation by ascorbic acid has also been reported to be more powerful in the presence of light and oxygen [269], which has no adverse effects on animal or human health and can be used easily.

Another natural protection was found for the combination of vinegar, sodium bicarbonate, and citric acid supplemented to apple juice, which has been reported to reduce PAT content [266,298].

A similar protection against OTA-induced immunotoxicity was also established for vitamin E supplementation in a dose-dependent manner in chicken feed, but this protection was only found to be applicable at low contamination levels of OTA below 2 ppm [218].

Some natural changes in the carbohydrate, protein, and fat content of the diet—such as low carbohydrate content or calorie restriction, high protein content, and low dietary fats—were also found to be partially beneficial against target mycotoxicoses such as AF toxicosis [267,299].

The toxic effects of mycotoxins can also be reduced by administering mycotoxin-contaminated forage to animals that are less sensitive to a particular mycotoxin, such as ruminants, which are less sensitive to OTA because of its hydrolysis in the rumen to the non-toxic substance ochratoxin α (OTα) [300].

Therefore, any kind of knowledge regarding mycotoxin metabolism or the routes of mycotoxin excretion and degradation is of particular significance for any kind of mycotoxin in any kind of animal in order to facilitate the finding of adequate possibility for reducing its toxicity.

### Mechanisms of Protection by Knowing Target Mechanisms of Mycotoxin Toxicity

Generally, the toxic effects of mycotoxins can be reduced if the specific mechanisms of toxicity are known for each target mycotoxin. This way, it will be easier to find some antidotes or vitamins that could prevent the specific mechanisms of such toxic effects when given as supplements to the diet [5]. In this regard, when the mechanisms of OTA toxicity are clear (for example, OTA suppression of energy metabolism, suppression of protein synthesis, or increase in lipid peroxidation), some proper antidotes that activate energy metabolism and protein synthesis or suppress lipid peroxidation could be experimentally evaluated for possible protection against OTA. For example, knowing that OTA toxicity is partly due to its structural homology with phenylalanine, which is responsible for protein synthesis suppression due to competition for a specific t-RNA [301], it could be assumed that phenylalanine would be a possible protector against OTA toxicity. However, the protective properties of phenylalanine against OTA-induced toxicity have been studied mainly via in vitro experiments, but few studies have been conducted with laboratory animals or poultry, suggesting partial protective effects of phenylalanine against OTA [6,10,11,14,15,209]. Therefore, such gaps in existing strategies need to be filled by conducting more “in vivo” studies to prove the suitability of such methods for protection against mycotoxins.

## 5. Novelties and Limitations of Biocontrol Approach Against Mycotoxins

### 5.1. Novelties and Advantages of Biocontrol Approach Against Mycotoxins

The high efficacy and lack of contamination in processed feed/food is a major advantage of the biocontrol approach and could explain its leading role as a promising new strategy for mycotoxin control.

Natural feed supplements usually attract the attention of industrial feed producers because they propose a safer and more cost-effective strategy for reducing mycotoxin exposure than other methods for mycotoxin decontamination [39]. Such supplements are non-toxic to animals and poultry [5,35,36,37] and can be easily excreted from the body [38]. The prevention of economic loss due to the scrapping of mycotoxin-contaminated feedstuffs or the decreased body weight of animals/poults exposed to mycotoxin-contaminated feeds can be restricted only by investing funds to purchase target herbal, microbial, or enzyme products.

Biocontrol strategy, e.g., microorganisms, yeast, enzymes, and natural substances, against mycotoxins is estimated to be a very friendly control approach compared with physical and chemical methods, both of which can deteriorate the nutrient value of feedstuffs or food commodities. A biocontrol approach could be used to metabolize, destroy, or deactivate mycotoxins into less or non-toxic compounds. Therefore, the large-scale industrial use of the biocontrol approach is highly desirable. Natural feed additives, natural organic binders, and bioactive supplements can be used for the safe utilization of mycotoxin-contaminated grains, feedstuffs, or foods without any subsequent health problems for animals or humans [5,35,36].

Mycotoxin degradation using microorganisms/fungi/yeasts revealed a new path for food/feed safety. Microorganisms or fungi can also be used as biopesticides, e.g., commercially available biopesticide Afla-guard [183]. Such a biopesticide and biocontrol approach was proved to be effective on peanuts [158], cottonseed [159], and corn [160].

The microbial agents and mycotoxin degradation enzymes have great potential for industrial applications in food/feed as well as in the fermentation industry [84,128,129]. For example, carboxypeptidase that can degrade OTA has been cloned and used to detoxify OTA [67]. However, more efforts have to be undertaken to clarify the toxicity of the resulting degradation compounds and the mycotoxin detoxification mechanisms in each particular case.

### 5.2. Limitations of Findings

Unfortunately, biocontrol methods have some well-known limitations. For example, mycotoxin biodegradation could be an effective approach, but it depends on multiple factors such as pH values, temperature, water activity, and nutritional source, which are of high importance for the antifungal efficiency of antagonists. Extensive studies and practical work are required to establish the optimal conditions for application of each biocontrol strain (fungi, yeasts or microorganisms) before its further application and commercialization

On the other hand, mechanisms involved in a mycotoxin’s biological control remain unclear. Much research has focused on the ability of microorganisms/fungi/yeasts to detoxify mycotoxins, but a better understanding of the enzymes involved and their underlying mechanisms is still needed. Therefore, further efforts are required to clarify the mechanisms responsible for the detoxification process and isolate the enzymes involved. Furthermore, the detoxification mechanism is important to be clarified in each particular case to prevent the simultaneous synthesis of other toxic metabolites during mycotoxin detoxification. In this regard, knowing the degraded compound’s toxicity is of particular importance because it can be either less or more toxic than the parent toxins. Methods for extracting enzymes from microorganisms/yeasts/fungi also need to be clarified and applied for large-scale use.

The elaboration of appropriate protectors for each mycotoxin requires in-depth knowledge of the specific mechanisms of its toxicity. In this regard, additional efforts are needed to discover and implement such protection in large-scale industrial applications.

Herbal and plant protection against deleterious effects of mycotoxins is always partial and cannot completely prevent their toxic effect on animals and birds. The legislative restrictions on the use of food and feed additives in the EU, which are based on safety assessment and technological need, should also be taken into account at the time of industrial application of each natural feed additive, e.g herb or plant.

## 6. Concluding Remarks and Future Perspectives

Biological methods of mycotoxin detoxification can ensure better food safety and preserve the flavor, organoleptic properties, and nutritional quality of treated feedstuffs and food commodities as compared to traditional chemical or physical methods. Moreover, such methods are environmentally friendly, easily available, and cost-effective compared with chemical or physical detoxification methods. Therefore, various adsorbents, clay binders, fungicides, microorganisms, herbal or plant additives, and enzymes are considered more desirable methods for mycotoxin decontamination and could be used as more practical feed additives for this purpose. However, further research is required to reveal their real potential compared with other methods for mycotoxin decontamination. Considering that clay binders are poorly efficient against most mycotoxins, except PAT and AFs, natural organic binders are highly recommended for the same purpose because of their good efficiency against multi-mycotoxin contamination of feedstuffs and their good biodegradability, which prevents possible environmental pollution.

The use of microbial antagonists was found to be a good alternative against conventional fungicides, and “detoxification by biotransformation” is considered a valuable new strategy for controlling mycotoxin contamination because of its high efficiency and lack of pollution. Many biological supplements provided as feed additives can promote mycotoxin degradation or biotransformation by target microorganisms, enzymes, yeast culture extracts, or natural antioxidants, which are less toxic or non-toxic and can be readily excreted or even utilized by animals/poultry. Some natural antioxidants used as feed additives have been proven to be very effective in postharvest fungal control and PAT inhibition, as well as in fungal control and/or inhibition of AFs and OTA. Such additives generally receive considerable attention from commercial feed enterprises because they provide a safe strategy to prevent mycotoxin exposure and reduce the bioavailability of mycotoxins. Considering that many mycotoxins co-exist in food commodities and feedstuffs, finding highly efficient strains that biodegrade or adsorb a large number of mycotoxins simultaneously should be a trend in future investigations.

Some herbs or herbal extracts, such as *Tinospora cordifolia, Glycyrrhiza glabra, Silybum marianum*, silymarin, *Withania somnifera*, etc., or target plants such as turmeric powder, or polyenzyme complements such as Roxazyme-G, could also be used in real-life practice together with target mycotoxin-binding agents to minimize the harmful effects of mycotoxin exposure through feed/food and to ensure better utilization of feedstuffs, greater body weight gain, and increased egg production in commercial animals or chickens. This approach could reduce economic losses from decreased animal production or the eventual condemnation of mycotoxin-contaminated fodder, as little expense would be incurred when purchasing the same herbs or herbal extracts. However, further efforts should be undertaken for their practical application in animal and chicken industries.

On the other hand, natural compounds in plant extracts or volatiles, and phenolic compounds such as isothiocyanates, which are naturally present in plants or apples, are found to be highly efficient in the control of fungal growth in fruits, and their application is completely safe. Some biologically active substances in plant extracts may also act as antifungal agents, such as flavonoids, silymarin, carotenoids, and polyphenols, inhibiting the growth of some fungi, such as *Aspergillus flavus*, and preventing AFs contamination of feed/food. Therefore, they serve as a practical way to prevent mycotoxin contamination of feed. In this regard, any piece of information about the metabolism and mechanisms of detoxification or the removal of any particular mycotoxin in each animal species or humans is essential to ensure the safe use of mycotoxin-contaminated fodder or food commodities without increasing the potential hazards and health issues. Additional research efforts should be undertaken to clarify the actual mechanisms of detoxification or degradation of mycotoxins, and to isolate the enzymes involved in such degradation.

## Figures and Tables

**Table 1:** Natural protection by biodegradation, biotransformation, or binding of mycotoxins by target microorganisms, yeasts, fungi, or enzymes.

Biodegradation or Binding by Microorganisms, Yeasts, Fungi, or Enzymes	Degradation/Detoxification or Binding Mycotoxins	Reference
**Microorganisms**		
*Lactobacillus rhamnosus*	AFs binding capability	[46,47]
*Lactobacillus plantarum*	PAT degradation capacity to hydroascladiol	[48]
*Lactobacillus acidophilus*	PAT and OTA degradation capacity	[49]
*L. sanfrancisco, L*. *plantarum, L. brevis, Saccharomyces cerevisiae* yeast strain	OTA degradation capacity (around 50–54%)	[50,51]
*L. brevis, L. plantarum, Oenococcus oeni*, *Leuconostoc mesenteroides, Pediococcus acidilactici* identified from wine or grape must	OTA degradation capacity	[52]
*Bifidobacterium bifidum, B. breve, Lactobacillus delbrueckii bulgaricus, L*. *casei, L. paracasei, L. johnsonii, L. rhamnosus, L. plantarum, L. salivarius*	OTA degradation capacity (around 30–97%) to non-toxic compound OTα	[53]
*Lactic acid bacteria* (LAB)	PAT removing capacity	[54]
*Alicyclobacillus* spp.	PAT degradation capacity in juice	[55]
*Actinobacterial strains,* e.g., *Streptomyces AT8, AT10, SN7, G10, PT1*	OTA degradation capacity (between 22% and 52%) and/or adsorption capacity (between 16% and 33%)	[56]
*Flavobacterium aurantiacum*	AFs removing capacity	[57]
*Lactobacillus kefiri,* *Acetobacter syzygii*	OTA, AFB1 and ZEA degradation capacity	[58]
*Oenococcus oeni* identified from wine	OTA degradation capacity	[59]
*Gluconobacter oxydans*	PAT degradation capacity to Z-ascladiol and E-ascladiol in juice from apples	[60]
*Eubacterium* BBSH 797 strain	DON degradation capacity to the non-toxic de-epoxy-DON	[61]
*Bacillus licheniformis* *CM21,* *Sl-1*	OTA degradation capacity (35–98%)	[62,63]
*Bacillus licheniformis*	AFB1 degradation capacity (around 74%)	[63]
*B* *acillus* *subtilis*	AFB1 degradation capacity (around 85%)	[63]
*Pediococcus parvulus UTAD 473*	OTA degradation capacity (80–90%) to non-toxic compound OTα	[64]
*Acinetobacter calcoaceticus* str.	OTA degradation capacity to non-toxic compound OTα	[65,66]
*Bacillus amyloliquefaciens ASAG1*	OTA degradation capacity (about 98%) to non-toxic compound OTα	[67]
*Brevibacterium casei, B. epidermidis, B. iodinum, B. linens*	OTA degradation capacity (100%) to non-toxic compound OTα	[68]
*Bacillus subtilis CW 14*	OTA degradation capacity (up to 97%)	[69]
*Stenotrophomonas nitritreducens, Eubacterium callanderi, Sphingomonas paucimobilis, S. asaccharolytica*	OTA degradation capacity (between 95% and 100%) to non-toxic compound OTα	[70]
*Eubacterium biforme MM11* identified from intestinal content of swine	OTA and AFB1 degradation capacity (between 77% and 100%)	[71]
*Cupriavidus basilensis ŐR16* str. identified from soil	OTA degradation capacity (100%) to non-toxic compound OTα	[72]
*Luteimonas* sp. CW574, *Silanimonas* sp. CW282, *Stenotrophomonas* sp. CW117, *Pseudomonas aeruginosa* N17-1, *Lysobacter* sp. CW239	OTA degradation capacity	[39]
**Yeasts and Fungi**		
*Trichosporon mycotoxinivorans* yeast strain	OTA and ZEA detoxification capacity	[73]
*Trichosporon mycotoxinivorans* yeast strain	ZEA degradation capacity to non-toxic compound ZOM-1	[74]
*Trichosporon mycotoxinivorans* yeast str. and *Eubacterium* BBSH 797	DON, ZEA and OTA in vivo degradation capacity	[61,75,76]
*Komagataella pastoris* yeast strain	FUMs detoxification capacity	[77]
Yeast strains *Metschnikowia pulcherrima M320, MACH1; Pichia guilliermondii M8, M29; Rhodococcus erythropolis AR14; Kloeckera lindneri GAL5*	OTA degradation capacity (between 26% and 84%)	[78]
*Phaffia rhodozyma* yeast strain *CBS 5905*	OTA degradation capacity (around 90%) to non-toxic compound OTα, and OTA adsorption capacity (around 23%)	[79]
*Kluyveromyces marxianus yeast strain C2,* identified from intestinal content of pigs	OTA degradation capacity (82–83%)	[39]
*Yarrowia lipolytica* yeast strain	OTA degradation capacity (around 88%)	[80]
*Trichosporon yeast strains DSM 14162, DSM 14156, DSM 14153, 178; Trichosporon mycotoxinivorans MTV, 115; Cryptococcus 118; Rhodotorula sp*. *DSM 14155,* 124	OTA degradation capacity (between 80 and 100%) to non-toxic compound OTα	[70,73,81]
*Saccharomyces cerevisiae* yeast strain	AFs binding capability	[82]
*Saccharomyces cerevisiae* *yeast strain*	PAT degradation capacity to Z-ascladiol and E-ascladiol	[83]
Yeast strains *Schizosaccharomyces pombe, Kloeckera apiculata, Saccharomyces cerevisiae, Candida pulcherima, Candida intermedia, Candida friedrichii, Cyberlindnera jadinii, Torulaspora delbrueckii, Lachancea thermotolerans*	OTA degradation capacity (between 25% and 84%) to non-toxic compound OTα	[84,85,86,87]
*Rhodosporidium paludigenum* yeast strain	PAT degradation capacity to less toxic compound desoxypatulinic acid	[88]
*Rhodosporidium kratochvilovae* strain LS11 and *Sporobolomyces sp*. yeast strain IAM 13481	PAT degradation capacity to less toxic metabolites, e.g., desoxypatulinic acid and ascladiol	[89,90]
*Yarrowia lipolytica yeast strain Y-2,* *Brevundimonas vermicularis B-1*	OTA degradation capacity (between 84% and 87%)	[91]
*Phoma* sp., *Mucor* sp., *Rhizopus* spp. 663, 668 and 710, *Trichoderma* sp. 639, *Trichoderma harzianum*, *Bacillus subtilis, Alternaria* sp. and some *Sporotrichum strains*	AFs degradation capacity is around 65–99%	[92,93,94,95]
Oyster mushroom *Pleurotus ostreatus*	OTA detoxification capacity	[45]
*Byssochlamys nivea* str. FF1-2	PAT degradation capacity	[96]
*Aspergillus. Japonicus AX35, A. carbonarius SA332, A. niger GX312*	OTA degradation capacity (between 83% and 99%) to non-toxic compound OTα	[97]
*A*. *niger M00120*	OTA degradation capacity (up to 99%) to non-toxic compound OTα	[98]
*A. tubingensis M074, M036*	OTA degradation capacity (up to 95%) to non-toxic metabolite OTα	[99]
*A. wentii, A. carbonarius, A. niger, A. Japonicus, A. ochraceus, A. fumigatus, A. clavatus, A. versicolor, Cladosporium sp., P*. *spinulosum, P*. *aurantiogriseum, Botrytis cinerea,* identified from grapes	OTA degradation capacity (up to 80%) to non-toxic metabolite OTα	[100,101,102]
*Rhizopus microsporus*, *R. stolonifer, R. oryzae, R. homothallicus*	OTA degradation capacity (up to 96.5%) to non-toxic compound OTα	[103]
*Aureobasidium pullulans AU34-2, AU18-3B, AU14-3-1, LS30*	OTA degradation capacity (between 75 and 90%) to non-toxic compound OTα	[104]
*Pleurotus ostreatus*	OTA degradation capacity (up to 77%) to non-toxic compound OTα	[105]
*Candida guilliermondii*	PAT degradation capacity	[106]
*Candida guilliermondii, Candida lusitaniae, Candida famata, Kloeckera spp., Cryptococcus laurentii, Rhodotorula glutinis* identified from Turkish grapes for wine	OTA degradation capacity	[107]
**Enzymes**		
Carboxypeptidase Y originating from *Saccharomyces cerevisiae*	OTA degradation capacity to non-toxic compound OTα	[108]
Carboxypeptidase A originating from bovine pancreas	OTA degradation capacity to non-toxic compound OTα	[109,110,111]
Carboxypeptidase originating from *Bacillus amyloliquefaciens, Acinetobacter sp. neg1, Phaffia rhodozyma*,	OTA degradation capacity to non-toxic compound OTα	[67,79,112]
Hydrolase originating from *A. niger*	OTA degradation capacity to non-toxic compound OTα	[113]
Lipase A originating from *A. niger*	OTA degradation capacity to non-toxic compound OTα	[114]
A crude metalloenzyme originating from *A*. *niger*	OTA hydrolyzation capacity	[115]
A crude enzyme Ancex	OTA degradation capacity	[109]
Protease A originating from *A. niger*	OTA degradation capacity to non-toxic compound OTα	[109]
CotA laccase originating from *Bacillus licheniformis ZOM-1*	AFs, ZEA, and AOH degradation capacity	[116]
Enzymes glucose oxidase and/or peroxidase	*Alternaria* mycotoxin AOH degradation capacity in fruits	[116,117]
Enzymes polyphenol oxidase and/or peroxidase	PAT degradation capacity in fruits	[118]

**Table 2:** Microorganisms, yeasts, fungi, or bioactive natural substances effective against target mycotoxin-producing fungi.

Microorganisms, Yeasts, Fungi, or Bioactive Natural Substances Effective Against Target Mycotoxin-Producing Fungi	Inhibition or Suppression of Fungal Development/Growth and Subsequent Mycotoxin Production of Following Fungi	Reference
**Microorganisms**		
*Bacillus subtilis* strains	Inhibition of fungal development of *Fusarium* strains and following production of FUMs.	[34]
*Bacillus* spp., e.g., *B. subtilis, B. megaterium, B. mojavensis, B. amyloliquefaciens*, *B. mycoides, B. pumilus, B. cereus*, and *B. mojavensis*	Reported as good biocontrol agents against AFs contamination.	[147,148,149,150,151]
*Bacillus megaterium*	Reported to prevent nearly 100% of AFs production in broth medium.	[148]
*Bacillus subtilis*	Reported to control the development of *Aspergillus parasiticus* (nearly 92%) and subsequent AFs production by up to 100%.	[149]
Apple dip treatment with suspension of microorganisms *Bacillus subtilis* or *Pseudomonas fluorescens*	Inhibition of fungal development of *P. expansum* at the time of cold storage and following PAT production in apples.	[170,171]
Fermented cell-free supernatants of *Paenibacillus chibensis* CECT 375, *Bacillus amyloliquefaciens* CECT 493, and *Pantoea agglomerans* CECT 850.	In vitro antifungal activities against OTA- and AFs-producing fungi due to high content of acetic acid, lactic acid, phenyllactic acid, and benzoic acid.	[172]
*Actinobacterial strains,* e.g., *Streptomyces* G10, ML5, and MS1	Inhibition of expression of target genes responsible for biosynthesis of OTA by *A*. *carbonarius*.	[56]
*Lactobacillus plantarum*	Inhibition of fungal development of *P. expansum* and *A. parasiticus,* and following production of PAT and AFs.	[135]
*Lactobacillus* (LAB), e.g., *L. delbrueckii, L. plantarum, L. reuteri, L. acidophilus, L. rhamnosus, L. paraplantarum, L. fermentum, L. casei* and *L. pentosus*	Reported to be effective towards AFs, but *L. plantarum* was found to be the most effective against AFs production.	[173,174,175]
*Lactobacillus plantarum* B4496, *Lactobacillus brevis* 207 and *Lactobacillus sanfranciscensis* BB12 isolated from fermenting cocoa	Reported to have good in vitro antifungal activities against OTA-producing fungi *A. niger*, *A. Carbonarius*, and *A. ochraceus*, with capabilities ranging from 15% to 67%.	[176]
*Pseudomonas fluorescens*	Suppresses the conidia germination of *A. flavus* by nearly 20%, in addition to the inhibition of AFB1 production (above 99%) in peanut medium.	[177,178]
*Pseudomonas chlororaphis* isolated from maize	Reported to inhibit the development of *A. flavus* by nearly 100%.	[152]
*Pseudomonas protegens* strain AS15 isolated from rice grains	Suppress up to 83% of AFs production, in addition to the suppression of the development of *A. flavus* (up to 68%).	[179]
*Pseudomonas syringae*	Inhibition of postharvest fungal development of *P. expansum* and *Botrytis cinerea* (gray mold and blue mold) on apples and following PAT production.	[131]
*Streptomyces* strains, e.g., *S. anulatus, S. yanglinensis, S. roseolus,* and *S. alboflavus*	Found to be very effective against aflatoxigenic fungi such as *Aspergillus flavus*.	[153,154,155]
Bacteria *Serratia marcescens* strain JPP1 isolated from peanut shells	Suppress AFs production by nearly 98%, and subsequent development of *A. parasiticus* by nearly 95%.	[180]
Bacteria *Nannocystis exedens*	Found to suppress significantly the growth of *A. flavus* and *A. parasiticus*.	[156]
**Fungi and Yeasts**		
Ascomycota yeast species (*Candida guilliermondii P3 and Pichia ohmeri 158*)	Inhibition of fungal development of *Penicillium expansum* and following PAT production.	[181]
Non-toxigenic strains, e.g., *Aspergillus flavus*	Displacement of mycotoxigenic strains by biocompetition and subsequent decrease in AFs levels in the feedstuffs or foods.	[140,141,182]
Non-toxigenic strains of *Aspergillus flavus* AF36	Displacement of mycotoxigenic strains by biocompetition and subsequent decrease of AFs production in cotton and peanut between 70% and 90%.	[146]
Non-toxigenic *A. flavus* strain NRRL21882 and *A. parasiticus* strain NRRL21369, and commercially available biopesticide Afla-guard (*A. flavus* strain NRRL21882).	Reported to be very effective biocontrol agents against AFs contamination in peanuts when applied in field conditions at preharvest time or in postharvest storage.	[183]
Non-toxigenic *A. parasiticus* applied in the field	Decreases AFs contamination during storage time.	[161]
Atoxigenic strain BN30	Reported as very effective in preventing AFs contamination of maize in Africa.	[184]
*A. flavus* strains AF051	Reported as very effective in decreasing AFs contamination in peanut fields in China by up to 99%.	[185]
Atoxigenic CT3 and K49 strains	Reported to decrease AFs contamination of maize by up to 65–94% in a four-year study	[145]
Atoxigenic AR100G, AR27, and AFCHG2 strains of *A. flavus*	Reported to decrease AFs contamination in groundnut fields in Argentina.	[186]
Atoxigenic *Aspergillus niger* strain FS10	Reported to decrease AFs production in the field.	[187,188]
Atoxigenic *Penicillium chrysogenum* strain RP42C	Suppresses the growth of toxigenic *Aspergillus* strains.	[189]
Yeast strains: *Kluyveromyces* spp., *Debaryomyces hansenii* strain BCS003, *Candida maltose*, *Pichia anomala*, *Saccharomyces cerevisiae* RC016, and *Saccharomyces cerevisiae* RC008	Suppress the growth of toxigenic *Aspergillus* strains and subsequent AFs production	[147]
*Trichoderma* spp.: *T. viridae, T. harzianum, T. Auroviride*, and *T. longibrachiatum*	Reported to be very effective against AFs production in the field at a rate between 50% and 80%.	[147,190]
*Trichoderma* spp.	Reported to be very effective against AFs contamination in sweet corn and groundnut by 65% and 57%, respectively.	[157]
*Rhodotorula glutinis* LS11	Inhibition of fungal development of *P. expansum* and following PAT production.	[90]
*Pichia ohmeri* 158	Inhibition of fungal development of *P. expansum* and following PAT production.	[191]
*Pichia caribbica* yeast	Inhibition of blue mold rot and following production of PAT in apples.	[192]
*Pantoea agglomerans CPA-1* and *Candida sake CPA-2*	Inhibition of fungal development of *P. expansum* and following PAT production.	[193]
*Torulaspora delbrueckii* and *Candida membranifaciens*	Inhibition of fungal development of *P. expansum* and following PAT production.	[194,195]
*Saccharomycopsis schoenii* predacious yeast	Suppression and biological control of fungal development of *P. expansum, P. Digitatum*, and *P*. *italicum* by true predation.	[196]
**Bioactive natural substances**		
Vanillic acid	Inhibition of fungal development of *Aspergillus* species and following OTA production.	[197]
Polyphenols, flavonoids, silymarin, and carotenoids	Inhibition of fungal development of *A. flavus* and following AFs production.	[198,199,200]
Target essential oils, e.g., clove oil and cinnamon	Lowering PAT content in apples.	[201]
Natural extracts of orange peel, cistus, and eucalyptus extract in a grape-based medium at concentrations of 10 and 20 mg/mL	Natural extracts of orange peel and cistus were found to have a good antifungal activity against the toxigenic *Aspergillus carbonarius* strain, whereas eucalyptus extract was reported to reduce OTA production by up to 85% at concentration 10 mg/mL with slight influence on fungal growth.	[202]
Plant extracts of target essential oils, e.g., oregano (*Origanum vulgare* subsp. *hirtum*), lavender (*Lavandula stoechas*), spearmint (*Mentha spicata*), and sage (*Salvia Fruticosa*), as well as some monoterpenoids, e.g., enchone, carvone, carvacrol, 1,8-cineole, terpinen-4-ol, and α-pinene	Inhibition of fungal development of *Fusarium oxysporum, P*. *expansum, A*. *terreus, Verticillium dahliae*, and mycotoxin production by the same species.	[203]
Lyophilized filtrates of *Lentinula edodes*	Stimulated production of antioxidant enzymes (e.g., glutathione peroxidase, superoxide dismutase, and catalase,) by *A. parasiticus* and inhibited AFs production by the same species.	[204]
Garlic vapor or extract exposure of apples	Inhibition of fungal development of *P. expansum* and following PAT production.	[205]

**Table 3:** Herbs, plants, vitamins, and natural bio-substances having good protective possibilities against the harmful effects of mycotoxins, which could be used as feed supplements.

Herbs/Plants, Vitamins, or Natural Bio-Substances	Protective Properties Against Mycotoxins in Experimental Animals or Poultry	Reference
**Herbs and Plants**		
Roxazyme-G (polyenzyme complement synthesized by “*Trichoderma*” fungi) given at 200 ppm to chicken feeds	-Increases OTA-induced suppression of body weight gain -Increases OTA-induced decrease in egg production -Decrease OTA-induced rise in serum levels of urea, creatinine, and glucose -Protection against OTA-induced kidney and liver damages -Protection against OTA-induced suppression of humoral immune response -Protection against OTA-induced damages in lymphoid organs, e.g., spleen, bursa of Fabricius, and thymus	[6,209]
Rosallsat (a plant extract of bulbus *Allii Sativi* and seminum *Rosae caninae*), at dose 0.6 mL/kg b.w. per day, given to chicken feeds	-Decreases OTA content in kidneys and liver -Suppresses OTA-induced lipid peroxidation -Protection against OTA-provoked kidney and liver damages -Protection against OTA-provoked damages in lymphoid organs, e.g., spleen, bursa of Fabricius, and thymus	[210]
5% total water extract of *Cynara scolymus L* (Artichoke) prepared as steam infusion and given to chicks in levels of 5 mL/kg.b.w. via feeds or drinking water	-Increases hepatobiliary excretion of OTA -Improves diuresis and increases urinary excretion of OTA -Decreases OTA content in kidneys and liver -Improves OTA-induced suppression of body weight gain -Increases OTA-induced decrease in egg production -Protection against OTA-provoked liver and kidney damages -Protection against OTA-provoked damages in lymphoid organs, e.g., spleen, bursa of Fabricius, and thymus -Anti-permeability and vasoconstrictive effects towards OTA-provoked edematous changes -Decreases OTA-induced rise in serum levels of urea, creatinine, uric acid, and glucose -Protection against OTA-induced suppression of humoral immune response	[6,17,208,209,210]
Sesame seed given at level 80,000 ppm to chicken feed	-Improves OTA-induced suppression of body weight gain -Increases OTA-induced decrease in eggs production -Improves OTA-inhibited protein synthesis -Protection against OTA-provoked kidney and liver damages -Protection against OTA-provoked damages in lymphoid organs, e.g., spleen, bursa of Fabricius, and thymus -Decreases OTA-induced rise in serum urea and creatinine -Protection against OTA-induced suppression of humoral immune response	[6,209]
*Silybum marianum* given at levels of 1100 ppm to chicken feeds or Silymarin introduced at 1% to chicken diet	-Protection against OTA-provoked liver and kidney damages -Protection against OTA-provoked damages in lymphoid organs, e.g., spleen, bursa of Fabricius, and thymus -Decrease OTA-induced rise in serum levels of uric acid -Decrease OTA-induced rise in serum enzyme levels of AST and ALT -Protection against OTA-provoked suppression in humoral immune response	[211,213]
Silymarin given at 10,000 ppm to chicken feeds	Ameliorates the immunotoxic effects induced by 1 ppm OTA	[218]
*Silybum marianum* given at levels of 10,000 ppm to chicken feeds or Silymarin at 600 mg/kg b.w.	-Improve AFs-induced suppression of body weight gain -Decreases AFs-induced rise in serum enzyme levels of ALT, AST and ALP -Protection against AFs-provoked liver damages -Improves feed conversion ratio in AFs-treated chicks	[219,220]
Silymarin given orally to rats at dose 200 mg/kg b.w. daily	-Protection against AFs-provoked diabetic nephropathy -Increases the renal activity of antioxidant enzymes	[221]
*S. marianum* extract given to rats at 600 mg/kg b.w. or Silymarin given to rats at 50 mg/kg b.w. or dogs at 20 mg/kg b.w.	-Protection against experimental damages in kidneys -Protection against the increase in lipid peroxidation -Protective effect against the increase in serum creatinine and urea	[222,223]
*Silybum marianum* given at various levels or Silymarin given to rats at dose 50–200 mg/kg b.w. per day	-Protection against experimental damages in liver -Protection against the increased serum levels of ALT, AST, ALP, γ-GT, and LDH -Suppresses lipid peroxidation in rats/mice -Protection against oxidative stress -Protection against carcinogenicity of various chemical agents	[224,225,226,227,228]
*Silybum marianum* or Silymarin studied in in vivo or in vitro studies	-Protection against humoral and cellular immunity -Antioxidative effect against oxidative stress -Protective effects against chemical carcinogenesis	[229,230,231]
*Withania somnifera* extract given at dose 500 mg/kg/day or Silymarin given at dose 150 mg/kg/day to rats	-Protective effect against liver damages -Suppressive effect on lipid peroxidation -Decreases serum enzyme levels of AST, ALT, and LDH -Antioxidative effect against oxidative stress	[232]
*Withania somnifera* given at levels of 4000 ppm to chicken feeds	-Protection against OTA-induced liver damages -Protection against OTA-provoked damages in lymphoid organs -Protection against OTA-induced suppression on humoral immune response -Decreases OTA-induced rise in serum enzyme levels of ALT and AST	[211]
*Withania somnifera* extract at dose 20 mg (dose per mouse i.p.)	-Protection of humoral and cellular immunity -Antioxidative properties	[233]
*Withania somnifera* extract given at dose 40 mg/kg b.w.	-Protection against brain damages	[234]
*Withania somnifera* given at various feed levels	-Improves body weight gain -Immunomodulatory properties -Antioxidative properties -Anti-neoplastic properties -Anti-inflammatory properties	[235]
*Centella asiatica* at different doses in different animals	-Protection of skin, vascular intima and gastrointestinal mucosa -Protection against oxidative stress -Antibacterial properties	[236,237]
*Centella asiatica* given at levels of 4600 ppm to chicken feeds	-Slight protective properties against OTA-induced suppression of humoral immunity -A slight protection against OTA-provoked damages in lymphoid organs -Decreases OTA-induced rise in serum enzyme activity of AST	[211]
*Tinospora cordifolia* extracts at different doses in different animals	-Antioxidative, anti-neoplastic, hepatoprotective, antidiabetic, and anti-inflammatory properties -Immunomodulatory properties -Suppression of lipid peroxidation -Diuretic properties	[238,239,240]
*Centella asiatica* essential oil	-Immunostimulating properties -Protection towards kidney and liver damages -Antibacterial properties	[241]
*Tinospora cordifolia* given at levels of 4000 ppm to chicken feeds	-Improves OTA-suppressed body weight gain -Protection against OTA-induced suppression of humoral immune response -Protection against OTA-induced kidney and liver damages -Decreases OTA-induced rise in serum levels of uric acid and glucose	[212]
*Tinospora cordifolia* extract given at 100 mg/kg b.w. per day to mice for 12 days	-Protection against OTA-induced changes in spleen and blood biochemistry in mice -Antioxidative properties against OTA-induced oxidative stress -Protection against genotoxic effect of OTA	[215,216]
*Tinospora cordifolia* at different doses in different animals or humans	-Protection against liver damages -Improves humoral and cellular immunity	[239,242,243,244,245]
*Tinospora cordifolia* extract in vitro study	-Antioxidative properties	[246]
*Tinospora cordifolia* extract given to mice at doses of 50–200 mg/kg b.w.per day	-Protection against AFs-induced oxidative stress -Protection against AFs-induced liver and kidney damages	[247]
*Tinospora cordifolia* at different doses in different animals or humans	-Protection of gastrointestinal mucosa -Protection against liver damages -Improves humoral and cellular immunity	[248,249,250,251,252]
*Tinospora cordifolia* extract given to rats at dose of 250 mg/kg b.w.per day	-Antidiabetic properties proven by suppression of alpha glucosidase activity	[253,254]
*Glycyrrhiza glabra* extract in vitro study	-Suppression of lipid peroxidation -Antioxidative properties	[255,256,257]
*Glycyrrhiza glabra* extract given to mice at doses of 750–1500 mg/kg b.w.per day	-Improves humoral and cellular immunity	[258]
*Glycyrrhiza glabra* extract given at dose of 2000 mg/kg b.w./day to rats or 50–200 mg/kg b.w. day to rats	-Hepatoprotective properties -Antioxidative properties -Decreases enzyme activities of ALT, ALP, and AST in serum	[224,259]
*Glycyrrhiza glabra* given at levels of 6600 ppm to chicken feeds	-Improves OTA-suppressed body weight gain -Decreases OTA-induced rise in serum enzyme levels of AST -Protection against OTA-induced liver damages -Protection against OTA-induced suppression of humoral immunity	[212]
*Glycyrrhiza glabra* given at different doses to rats	-Protection of liver -Lipid-lowering action -Decrease cholesterol -Inhibition of lipid peroxidation	[260]
*Glycyrrhiza glabra* at different doses in different animals	-Antibacterial/antiviral properties -Anti-inflammatory properties -Anti-hyper glycemic properties	[261]
Polyherbal additive “Growell” given at 350 or 750 ppm to chicken feed	-Protection against AFs- or OTA-provoked blood biochemical changes -Protection against AFs- or OTA-provoked pathological changes in internal organs, e.g., liver, kidney, spleen, bursa of Fabricius, and thymus of broilers	[262,263]
*B. refescens, A. leiocarpus, I. asarifolia, G. senegalensis and M. oleifera*	-Antioxidative properties	[264]
Turmeric powder given at 400 ppm to chicken feed	-Antioxidative properties in broilers -Protection against AFB1-provoked increase in lipid peroxidation -Decreases AFB1 contamination levels in liver of broilers up to undetectable levels	[265]
**Vitamins or natural bio-substances**		
Phenylalanine given to mice, rats, or chicks at levels 20–25 ppm to the feeds	-Improves OTA-induced suppression of body weight gain -Improves OTA-induced suppression of eggs production -Improves OTA-induced suppression of protein synthesis -Protection against OTA-provoked kidney and liver damages -Protection against OTA-provoked damages in lymphoid organs, e.g., spleen, bursa of Fabricius, and thymus -Decreases OTA-induced rise in serum urea and creatinine -Protection against OTA-provoked suppression of humoral immune response -Protection against OTA-provoked carcinogenic effect in rats or chicks -Protection against OTA-provoked teratogenic effect in mice	[6,10,11,14,15] [209]
Citric acid addition to apple juice	-Decreases content of PAT in apple juice	[266]
Ascorbic acid and/or vitamin B addition in vivo or in vitro studies	-Decreases content of PAT in apple juice	[267,268,269,270]
Ascorbic acid addition at 300 ppm to the diet of laying hens	-Protection against OTA-provoked decrease on egg production, eggs shell damages, and decrease in eggs’ weight	[271,272]
Vitamin E supplementation at 200 ppm to the cockerels’ diet	-Protection against OTA-provoked immunosuppression	[218]
Ursolic acid	-Protection against OTA-induced kidney damages -Antioxidative properties against OTA-induced oxidative stress -Reducing the apoptotic effect of OTA -Protection against OTA-induced decrease in cell viability of human embryonic kidney 293T (HEK293T) cells	[273,274]
Oleanolic acid	-Protection against OTA-induced kidney damages -Amelioration of OTA-induced apoptotic damages -Increased viability of OTA-treated HK-2 cells	[275]

## Data Availability

The original contributions presented in the study are included in the article, further inquiries can be directed to the corresponding author.
